# A Dynamical Model of Hierarchical Selection and Coordination in Speech Planning

**DOI:** 10.1371/journal.pone.0062800

**Published:** 2013-04-24

**Authors:** Sam Tilsen

**Affiliations:** Department of Linguistics, Cornell University, Ithaca, New York, United States of America; McMaster University, Canada

## Abstract

Studies of the control of complex sequential movements have dissociated two aspects of movement planning: control over the sequential selection of movement plans, and control over the precise timing of movement execution. This distinction is particularly relevant in the production of speech: utterances contain sequentially ordered words and syllables, but articulatory movements are often executed in a non-sequential, overlapping manner with precisely coordinated relative timing. This study presents a hybrid dynamical model in which competitive activation controls selection of movement plans and coupled oscillatory systems govern coordination. The model departs from previous approaches by ascribing an important role to competitive selection of articulatory plans within a syllable. Numerical simulations show that the model reproduces a variety of speech production phenomena, such as effects of preparation and utterance composition on reaction time, and asymmetries in patterns of articulatory timing associated with onsets and codas. The model furthermore provides a unified understanding of a diverse group of phonetic and phonological phenomena which have not previously been related.

## Introduction

### Coordination and selection in motor planning

Many of the motor activities we perform from day-to-day involve sequences of movements in which some of the movements are initiated with precise timing. Past research on motor control has established an important distinction between the sequencing of movements and the coordination of their timing [1]–[4]. For example, consider the task of making a 90° turn in an automobile at an intersection. The driver of the vehicle will press the brake pedal to decelerate, then rotate the wheel and release the brake pedal. At reasonable speeds, the deceleration and rotation are typically performed in sequence, whereas the release of the brake is usually contemporaneous with the rotation of the wheel, i.e. these movements are initiated at approximately the same time. When we observe a precisely controlled temporal relation between movements, we say that they are *coordinated*. This precision control has important consequences for accomplishing the goals of the task. If the driver were to substantially delay one or the other movement, they could easily crash or damage the automobile. Hence we distinguish between two aspects of motor control: sequencing, the control of the order in which movements are performed, and coordination, precision control of the relative timing of movement initiation.

Several models of motor planning have understood sequencing with the concept of *competitive selection*: movement plans are activated in parallel prior to initiation, and then they are selected in order through mutually exclusive competitive processes [5], [6]. This understanding works well as long as motor plans are viewed at a sufficiently abstract level of a planning hierarchy. For example, we can consider rotating the wheel and releasing the brake to be subcomponents of a more abstract “turn” program. At this abstract level, we can view the task as consisting of two sequentially selected motor programs: brake and turn. In this case, the precisely coordinated movements of rotating the wheel and releasing the brake are *co-selected* subcomponents of the turning program.

One puzzle for theories of motor planning is how selection and coordination interact. While there currently exist models of motor planning based on competitive selection and models of coordination, no model integrating both of these mechanisms has yet been developed. This paper presents a dynamical model of motor planning and execution – the *activation-spin model* – that integrates both coordination and selection mechanisms. The model focuses on the articulatory movements of speech and more abstract speech units such as syllables and words, which together can be viewed as parts of a hierarchical structure of motor plans. The model is an integration and elaboration of two different frameworks, articulatory phonology and competitive queuing. Articulatory phonology is a theory of phonological representation and phonetic implementation that utilizes phase-coupling forces between oscillatory systems to govern articulatory timing. Competitive queuing is a general dynamical framework for sequential selection of simultaneously active motor plans, which can be readily applied to the word and syllable components of utterances.

A key innovation of the model developed here is that it attributes an important role to selection in the control of articulatory timing *within a syllable*. Selection has been investigated primarily with regard to the word and syllable levels of the speech motor hierarchy, and coordination with regard to the level of articulatory gestures. The most obvious approach to integrating coordination and selection would apply these mechanisms to the levels of the motor planning hierarchy with which they have traditionally been associated. In other words, a null hypothesis would hold that competitive selection governs the sequencing of words and syllables, whereas coordinative mechanisms govern the timing of articulatory gestures. In such a view articulatory gestures would be selected automatically when their associated syllable is selected. In contrast, the activation-spin model proposes that competitive selection plays a crucial role in the control of articulation within a syllable. Specifically, the model holds that onset consonantal gestures and vocalic gestures are co-selected (i.e. not competitively selected) and coordinated, while coda consonantal gestures are competitively selected relative to a preceding vocalic or consonantal gesture, rather than coordinated. This constitutes a substantial departure from the coupled oscillators model of articulatory phonology, because it restricts the involvement of coordination in the control of articulatory timing. Hence the proposal here is not merely a trivial combination of previously proposed mechanisms, but an integration that attributes a novel role to selection in controlling articulatory timing.

The proposed integration is significant because it offers a unified account for a variety of phonetic and phonological asymmetries between onset and coda consonants. Most phonological theories take for granted a hierarchical organization of sounds into syllables. A syllable can be analyzed to consist of an onset (consonants preceding the vowel), a nucleus (the vowel itself), and a coda (consonants following the vowel). When multiple consonants occur in onset or coda position, the syllable is said to have a *complex onset* or *complex coda*. There is a diverse collection of cross-linguistic typological differences between onsets and codas, which include the following: (1) onsets combine relatively freely with vowels, whereas certain combinations of codas and vowels are more commonly restricted [7], [8]; (2) codas often function to fulfill templates for morpheme or syllable structure, whereas onsets do not [9]; (3) in languages with lexical tone, onset consonants rarely influence the capacity of a syllable to bear certain types of complex or contour tones, whereas the presence of a coda consonant can influence the tone-bearing capacity of a syllable [10], [11]; (4) in languages with stress, the presence of a coda consonant can influence the location of stress within a word, whereas onsets do not exhibit this influence [12], [13]; (5) on diachronic timescales, the reduction of articulatory gestures in a coda consonant can induce a preceding vowel to be lengthened – a pattern known as compensatory lengthening, whereas the loss of an onset consonant never results in lengthening of an adjacent vowel [12], [14]; (6) in accelerating syllable repetition tasks, VC (vowel-coda) syllables spontaneously reorganize into CV (onset-vowel) syllables, whereas the reverse does not occur [15], [16]. These are just some of the numerous differences between onset and coda consonants, all of which beg for a unified explanation. Below we evaluate how the standard coupled oscillators model and the proposed activation-spin model account for these differences. We conclude that the proposed integration of coordination and selection mechanisms offers a more comprehensive explanation of the above asymmetries.

Before presenting the activation-spin model, we review below basic aspects of its precursors – the coupled oscillators model of articulatory phonology and the competitive queuing model of sequential selection – along with the key behavioral phenomena these account for. It should be noted that, despite integrating two important theoretical frameworks, the scope of the model presented here is relatively limited: the current implementation focuses on the planning and initiation of speech movements in a hierarchical structure of syllables and words; the model does not address a number of equally important issues, such as motor learning, the role of feedback in control, or the control of effector trajectories. Despite these limitations, the activation-spin model is an important development because it explains a broad range of behavioral phenomena related to sequencing and movement timing.

### Articulatory phonology and the coupled oscillators model of coordination

Many theories of phonology have assumed that the basic cognitive units of speech are discrete, symbolic units such as segments or features. However, attempts to identify invariant acoustic correlates of these units in recordings of speech have been unsuccessful. Articulatory phonology addresses this problem by proposing that articulatory gestures, rather than segments, are the basic units of speech [17], [18]. In this framework, articulatory gestures are conceptualized as dynamical events in which organs of the vocal tract move to achieve constriction targets. The targets themselves are defined in coordinates of vocal tract geometry [19], such the aperture between the lips or the location and degree of a constriction between the tongue and the palate. For example, in the word “spa” the consonant/s/ is associated with a narrow constriction gesture near the anterior of the palate made by raising the tongue tip; the consonant/p/ is associated with a bilabial closure gesture, in which the aperture between the lips is closed to prevent airflow; and the vowel/a/ is associated with a lowering and retraction of the tongue body to create a narrowing along the rear wall of the pharynx, thereby influencing the resonances of the vocal tract in a distinctive way.

A key tenet of articulatory phonology is that gestures can overlap in time. This possibility allows for a large amount of contextual variation in the acoustic signal to be explained. One concrete example is the observation that the location of the closure between the tongue and the palate in/ki/ is more anterior than it is in/ku/ – this pattern can be readily understood to result from the coproduction of the consonant and vowel, which can be modeled as the simultaneous activation of both gestures [19]. This sort of variation is not easy to accommodate in models of phonology based on linear sequences of units. In contrast, articulatory phonology can more readily account for overlap because it incorporates the relative timing (i.e. coordination) of gestures directly into lexical representations.

**Figure 1:**
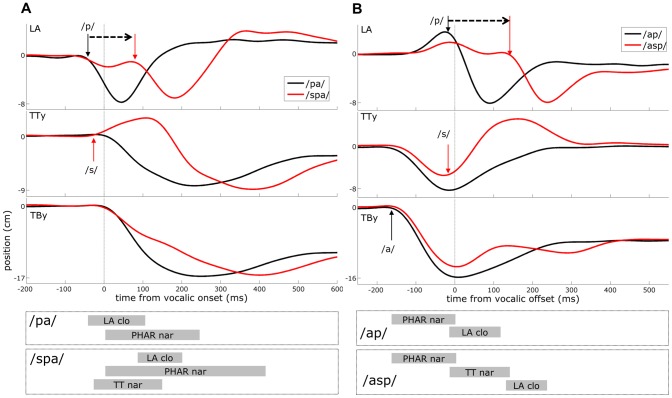
Illustration of articulatory coordination and sequencing. (A) onset consonantal gestures are precisely coordinated with the vocalic gesture and overlap substantially. (B) coda consonantal gestures are selected sequentially. Arrows show movement onsets. Gestural scores aligned to movement trajectories are shown for consonantal and vocalic gestures.

An important observation in studies of speech articulation is that the relative timing of consonantal and vocalic gestures depends on the position of the consonant within the syllable. Onset consonants are consonants that occur before a vowel in a syllable, and coda consonants occur after the vowel. Articulation studies have shown that onset consonantal gestures and vocalic gestures tend to be initiated closely together in time, whereas coda consonantal gestures are initiated at the offset of the preceding vocalic gesture. This *onset/coda asymmetry* is illustrated in Figure 1, which shows articulatory movements recorded with electromagnetic articulography [20]. Figure 1A shows the case in which consonantal gestures precede a vowel, here the consonant-vowel (CV) form/pa/ and the CCV form/spa/. The panels show the following from top to bottom: distance between upper and lower lip sensors, i.e. lip aperture (LA); vertical position of the tongue tip/blade (TTy); and vertical position of the tongue body/dorsum (TBy). Below the movement trajectories are gestural scores, which represent periods of gestural activation. In the task-dynamic model of speech production [19], gestural activation corresponds to a period of time in which a movement intention is present in the form of the displacement of a vocal tract equilibrium from its neutral value. The articulatory movements shown here were produced in response to a go-cue given in the midst of a sustained vowel/i/, which is produced with a relatively high position of the tongue dorsum.

To observe the temporal proximity of the initiation of the onset consonant and vowel gestures, notice that in the CV form/pa/, the bilabial closure for/p/ (a decrease of LA) begins nearly at the same time as the vocalic/a/gesture that lowers and retracts the body of the tongue (TBy) – the delay between these movement onsets is only about 50ms. Moreover, the periods of time in which these two movements occur overlap extensively. The same is true for/spa/, where the raising of the tongue tip (TTy) for/s/ precedes the initiation of the vocalic gesture by about 30 ms and the initiation of the bilabial closure follows the vocalic gesture by about 80 ms. These patterns indicate that consonant and vowel articulatory gestures are precisely coordinated when the consonants are onsets of a syllable.

The onset consonant timing pattern contrasts markedly with the pattern observed for coda consonants in the forms/ap/and/asp/. Figure 1B shows that in a coda consonant, the articulatory movement is initiated near the *offset* of a preceding gesture. In the form/ap/, the bilabial closure is initiated as the preceding vocalic movement reaches its target, and in the complex coda form/asp/, this gesture is initiated near the offset of the preceding consonantal gesture for/s/. Hence the coda/s/ and/p/ gestures appear to be sequentially timed relative to a preceding gesture: first the/a/ gesture is selected and executed, and then the following/s/consonantal gesture is selected and executed, and then the following/p/ gesture is selected and executed. In other words, the coda articulations are not precisely coordinated with the initiation of the vowel articulation as they are in the CV and CCV forms.

**Figure 2:**
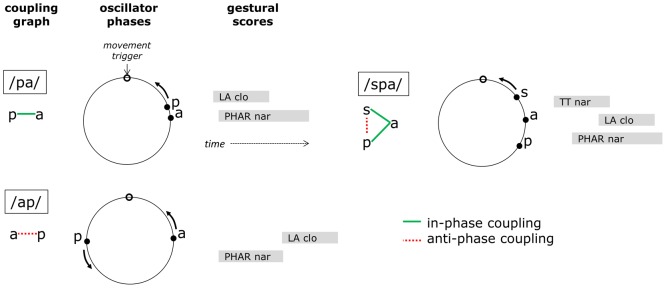
Coupled oscillators model of coordination for CV (/pa/), CCV (/spa/), and VC (/ap/) forms. For each form, a coupling graph is shown with in-phase coupling (solid green lines) and anti-phase coupling (dashed red lines). Oscillator phases proceed counter-clockwise until they reach an arbitrary phase value that triggers movement initiation, i.e. activation of the corresponding gesture in a gestural score.

Articulatory phonology accounts for the onset/coda asymmetry by conceptualizing the syllable as a system of coupled gestural planning oscillators. In early versions of the framework intergestural timing relations were specified directly in lexical representations. Building upon the dynamical model of movement coordination in [21], the innovation in [22] introduced a dynamics of planning that computes intergestural timing from a network of phase-coupled oscillators. In this system, each articulatory gesture is associated with a planning oscillator, and the planning oscillators may be coupled to one another in one of two ways: they may be in-phase coupled, such that coupling forces minimize the phase difference of the oscillations, or anti-phase coupled, such that coupling forces maximize the phase difference. By hypothesizing that onset consonantal gestures are in-phase coupled to a vocalic gesture, this model accounts for the empirical observation of nearly synchronous movement initiation. As schematized in Figure 2, the dynamic phase variable of each planning oscillator can be viewed as an angle made by a point moving counterclockwise on a unit circle. The oscillators are assumed to maintain a constant amplitude, exhibit 1:1 frequency-locking of their intrinsic phase velocities, and depending on their initial conditions, require time to evolve toward a stable relative phase pattern prior to the triggering process. The gesture corresponding to each planning oscillator is triggered (initiated) at an arbitrary phase angle, here the top of the circle. For a simplex onset as in/pa/, the consonantal gesture is initiated at nearly the same time as the vocalic gesture because their associated planning oscillators are nearly in-phase.

Furthermore, by hypothesizing that in complex onsets (i.e. CCV or CCCV) consonantal gestures are anti-phase coupled to one another while simultaneously in-phase coupled to the vocalic gesture, the model can account for another important empirical pattern known as the c-center effect [23]. Studies in a number of languages have shown that as additional consonants are added to a syllable onset, the midpoint of the sequence of consonantal articulations maintains a fixed temporal relation to the vowel [24]–[27]. Accordingly, the timing of the rightmost (immediately pre-vocalic) consonant will shift closer to and overlap more with the vocalic gesture. The c-center effect is evident in the movement trajectories and gestural score of Figure 1A, and is portrayed schematically in Figure 2. When two onset gestures are present (e.g. in “spa”), the anti-phase coupling force between the consonantal gestures results in a temporal displacement of their respective triggering events relative to the vowel. In a sense, the co-existing in-phase and anti-phase coupling forces compete, and the stable relative phase of the system reflects a compromise between them.

One compelling basis for hypothesizing different coupling modes for consonant-vowel and consonant-consonant coordination relates to the interaction between the perception of articulatory gestures and mechanical coupling inherent in articulation. Constrictions made with the front and back of the tongue can interfere with one another in a mechanical sense, and also can interfere with labial constrictions through mutual mechanical coupling to the jaw. Moreover, when two consonantal constrictions are made contemporaneously, the more anterior one is liable to mask the acoustic consequences of the more posterior one. Hence if two consonantal articulations are produced simultaneously, they are likely to interfere and jeopardize the perceptual recoverability of the articulations. Hence from the standpoint of a listener, anti-phase coordination of consonants is preferable, because it minimizes temporal overlap between consonantal articulations. In contrast, vowel gestures achieve their targets more slowly than consonantal ones and involve a lesser degree of constriction; hence a vocalic gesture can be initiated simultaneously with a consonantal gesture without rendering the consonantal articulation perceptually unrecoverable [7]. In contrast, the achievement of a vocalic target, which occurs well after initiation of the movement, is susceptible to mechanical interference from a simultaneously produced consonant. Although coda consonants could in theory be in-phase coordinated with vowels and be produced even more slowly than either onset consonants or vowels, this would result in mechanical interference between the gesture for attaining the vocalic target and hinder its perceptibility, hence in-phase coordination between a vowel and coda consonant is undesirable.

The coupled oscillators model accounts for coda coordination by hypothesizing that coda consonantal gestures are anti-phase coupled to a preceding gesture. Hence in a VC_1_C_2_ syllable, the C_1_ gesture is anti-phase coupled to the preceding vowel, and the C_2_ gesture is anti-phase coupled to the preceding consonant. Figure 2 illustrates the case of a VC syllable/ap/, where the coda gesture is triggered 180° out of phase with the vocalic planning oscillator. This hypothesis receives some indirect support in the observation that coda gestural timing is more variable than onset timing, which could be due to the relative instability of anti-phase coupling compared to in-phase coupling [28]. However, because gestural planning oscillations are not directly observable in articulatory or acoustic data, and because the model predicts no analogue of the c-center effect for complex codas, only indirect evidence of this sort can be provided for the coda coordination hypothesis.

Furthermore, the articulatory phonology treatment of codas encounters a dilemma in explaining the observation that coda movements appear to be initiated at the offset of vocalic movements (see Figure 1B). First, consider that the model explicitly dissociates gesture durations from planning system periods. The planning systems serve the purpose of determining when gestures become active, but the durations of those activation intervals are independently specified. This is necessarily the case, given that all oscillators have a uniform intrinsic frequency and yet consonantal and vocalic movements can differ substantially in duration [29], [30]. Now consider the observation that there exists a close relation between the attainment of the vocalic target and the onset of the coda consonantal gesture, as is evident in the movement patterns for coda gestures shown in Figure 1B. Under the hypothesis of anti-phase coda coupling, in which the coda consonantal gesture is triggered 180° out of phase with the vowel, one might be tempted to assert that the duration of the vocalic gesture is precisely half of the period of its corresponding planning oscillator. This would naturally suggest that gestural durations are equal to half-periods of planning oscillators. Yet we know that this cannot hold true because of the aforementioned variation in gestural durations across segment types and syllable positions. Hence the phase-coupling model of coda coordination is left with no straightforward way to explain the basic observation that coda gestures appear to be initiated at the offset of vocalic gestures.

The coupled oscillators model of articulatory phonology does well in accounting for the coordination of onset consonants and vowels, but has a number of limitations in the scope of phenomena it explains. For one, there is the aforementioned dilemma in explaining why coda consonantal gestures are initiated near the attainment of vowel movement targets. More importantly, there are a number of phenomena involving larger units of linguistic organization – syllables, words, etc. – that a speech production model would ideally accommodate. As we discuss below, it is known that the number of words in an utterance and the number of syllables in those words have effects on how quickly the utterance can be initiated. Although there have been several extensions of the coupled oscillatory systems approach to incorporate planning dynamics associated with syllables, feet, and phrases [31]–[36], it is not clear how these models can account for such phenomena. Below we consider a different class of model that is designed to account for factors influencing utterance initiation.

### Sequential selection and competitive queuing

Early theories of the control of sequential movement were based on the concept of an *associative chain*. In this conception, motor units correspond to groups of motor neurons. One unit activates, effecting a motor response; sensory feedback from that response would activate the next unit in the chain, which would in turn effect a motor response and more feedback, activating the next unit, and so on. In a classic paper, Lashley argued that this view is untenable to explain behaviors involving serially ordered movements [1], taking particular objection to the assumption that units sequentially go from a quiescent state to an activated state and back again without substantial overlap in periods of activation. His arguments were based on the free combinability of units, the potential for associations to develop between non-adjacent elements in a sequence, and the occurrence of sequential errors. Lashley suggested an alternative view in which units are hierarchically organized by a central planning mechanism. Crucially, this mechanism would excite units prior to their execution, and it is the pattern of excitation which directs the control of serially ordered movements. In modern terms, this means that units in a sequence of movements are planned *in parallel*.

Effects of sequence length and unit complexity on temporal aspects of movement provide an important body of evidence for parallel planning [37]–[39]. In a series of experiments, Sternberg and colleagues demonstrated that the reaction time to initiate an utterance or typed sequence increases with the length of the sequence (e.g. the number of words) and complexity of the units (e.g. the number of syllables in each word). These effects are linear and additive, suggesting that the number of units within a given level of a motor response hierarchy (e.g. within word- and syllable-levels), influences response behavior. Moreover, the durations of the elements in the sequence are likewise increased by length and complexity. These findings are readily understood if movements are simultaneously planned and interact competitively so as to slow the time-course of selection.

Errors in sequencing provide a second body of evidence for parallel planning. Studies of speech errors suggest that units are active prior to, during, and subsequent to their production [40]–[42]. Regarding errors involving segmental units, these studies have shown that both anticipatory and perseveratory errors occur. Anticipatory errors involve the errorful production of a segment or group of segments prior to its expected location in a sequence, e.g. “[pl]*eech planning*”. Perseveratory errors involve the errorful production of a segment subsequent to its expected location in a sequence, e.g. “*speech* [sp]*anning*”. Sometimes these co-occur resulting in an exchange (spoonerism): “[pl]*eech* [sp]*anning*”. A reasonable conclusion is that error patterns of this sort could only arise if errorful units are activated in planning well prior to and subsequent to their temporal location in a sequence.

Models of sequential movement planning have been developed to account for the effects of sequence length and complexity on reaction times, as well as the commonly observed anticipatory and perseveratory error patterns. A key idea in many approaches is the concept of *motor plan selection*, which is a process that dissociates anticipatory activation of motor programs from mechanisms of selection and execution. One example is the sequential selection model of Sternberg et al. [39]. Motor programs are stored in a memory buffer, then subsequently selected by a search mechanism that takes longer if there are more programs in the buffer. Subsequent to selection a motor program is executed and then re-enters the memory buffer. The tripartite division between activation, selection, and execution is a common theme in other approaches, including models which incorporate continuous activation dynamics.

**Figure 3:**
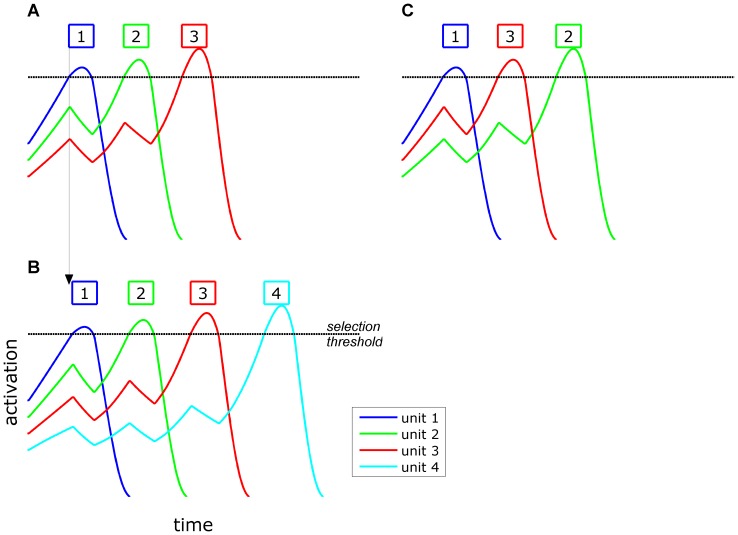
Competitive selection model dynamics. (A) competitive selection of three units. (B) competitive selection of four units, with movement initiation delayed relative to (A). (C) Sequencing error in which unit 3 is selected early.

An important advance in modeling sequential movement planning was developed in [5], which introduced the concept of *competitive-queuing* of action units. In this approach (see Figure 3), motor units are associated with activation variables. In a pre-selection planning stage, the motor plans become activated to varying degrees. The intention to initiate the movement sequence results in growth of activation and selection of the most highly active plan, which triggers its execution. The selected plan is subsequently suppressed (either directly through recurrent self-inhibition or indirectly through sensory-feedback mechanisms), and this allows the next most highly active plan to be selected, suppressed, and so on. A consequence of this design is that the relative activation of motor plans determines the order in which they are selected, and hence such models can simulate sequential selection. A number of models with a similar activation-dependent selection mechanism have been developed [6], [43], [44].

Competitive queuing dynamics are successful in accounting for effects of sequence length and unit complexity, as well as common error patterns. In such models, latency to select a motor plan depends upon the time for activation levels of that motor plan to win the competition for selection. Having more plans simultaneously active decreases the activation level of each plan, either due to inhibitory interactions between active plans or due to normalization of total activation. When a sequence contains more movements, the activation of each movement plan will be diminished by the presence of more inhibitory interactions and hence take longer to be selected. The predicts the aforementioned empirical observations: longer RTs and unit durations in longer movement sequences. This effect can be observed by comparing Figure 3A and 3B: the latency of initiation of the first unit is greater when there are more plans, and the intervals between selections of plans likewise increase. It is also straightforward to generate common error patterns in such models. If for some reason the relative activation pattern is altered early on, perhaps by noise or external influences, selection will proceed in an errorful way (Figure 3C). Dell showed that anticipatory errors can arise in this way, and that perseveratory errors can occur when a plan fails to be fully suppressed and is subsequently reselected [43].

Another phenomenon amenable to explanation in a competitive selection model is the dependence of RT on response preparation. In delayed naming tasks, the target response is known well in advance of the response cue, and hence response plans are active prior to their selection. In contrast, in immediate response tasks, response plans are given by the response cue itself, and hence the latency to initiate the response includes both retrieval and selection of the response plans. Part of this effect is due to competition in retrieval: more frequent syllables and more phonotactically probable segmental sequences are initiated more rapidly [45]–[47]. Reaction times based on articulatory movement initiation in a prepared response paradigm have been reported to be approximately 180 ms for stop-initial CV syllables [48]. Crucially, that study controlled pre-response articulatory posture to prevent speakers from configuring their vocal tract to facilitate rapid reaction time, which [49] found to be a common strategy. In contrast, RTs in an unprepared response task are substantially longer. While no studies have directly compared prepared and unprepared CV response RTs with the appropriate controls on pre-response posture, there are several studies which suggest that the additional delay in an immediate response task is on the order of 150–250 ms [49]–[51]. Sequential selection models can readily account for preparation effects by associating preparation with heightened activation prior to the intention to initiate a response. In unprepared responses, activation takes longer to reach the selection threshold because activation values begin relatively low; in prepared responses, activation values begin relatively high, and hence the selection threshold can be reached more rapidly.

The timing of articulatory movements, however, does not always conform to predictions made by strictly sequential models of selection. As observed above, onset consonantal gestures in CV and CCV forms are initiated quite closely in time with respect to the vocalic gesture, and these consonantal movements overlap extensively with the vocalic movement. This suggests that selection of the vocalic gesture is not delayed until the deselection of a preceding consonantal gesture. Studies of the effects of syllable structure on the reaction time to initiate a speech response also support the notion that onset consonants and vowels are not sequentially selected. Recall that a key prediction of selection models is the length effect: the reaction time to initiate a motor program increases with the number of units in the program. For example, if V, CV, and CCV responses are held to consist of one, two, and three units, respectively, a sequential selection model would predict that the RT to initiate a response should increase from V to CV to CCV. Yet this prediction has been not been upheld. Experiments reported in [48], [52] have found either no difference between latencies in CV and CCV responses, or have found certain CCV responses to be initiated more rapidly than CV responses (this latter, unexpected effect is observed in/sC/ onset clusters and can probably be explained by conditional probability in orthographic stimuli [52]). Hence the absence of length effects suggests that competitive selection is not sufficient for understanding movement planning at the level of articulatory gestures.

### The activation-spin model: integrating selection and coordination

The available evidence indicates that there is an important distinction between how onset and coda consonantal gestures are produced: movements associated with onset consonants are co-selected and tightly coordinated with vocalic movements; in contrast, movements associated with coda consonants are sequentially selected relative to a preceding movement. Furthermore, the number of words and syllables in an utterance has an effect on the RT to initiate the utterance, as does the extent to which the response has been prepared. Neither a strictly sequential selection mechanism nor a coupled oscillators coordination mechanism can fully account for all of these patterns. To address this problem, a hybrid model – the *activation-spin model* – is developed here, which integrates both selection and coordination mechanisms.

**Figure 4:**
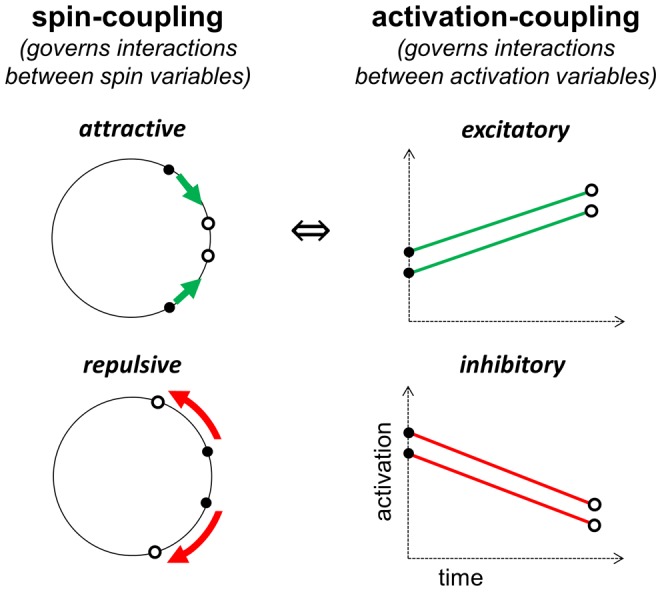
Schematic illustration of effects of spin-coupling and activation coupling. Spin-coupling forces act to decrease or increase the phase difference between oscillatory systems. Activation-coupling forces act mutually to increase or decrease activation.

The model utilizes two main dynamical variables, activation and spin, and accordingly two types of coupling forces, activation-coupling and spin-coupling. The former regulates how activation variables interact, and the latter how spin variables interact. Each articulatory gesture, syllable, and word in the hierarchical structure of an utterance is associated with a distinct *planning system*, and for each planning system there is an activation variable and a spin variable. Previous efforts to integrate selection and coordination attempted to model these mechanisms using just a single variable that exhibited both oscillatory and competitive-selection dynamics [36], [53], [54]. However, strong interactions between oscillation- and selection-related changes in activation created a variety of difficulties. The use of two distinct variables is a key innovation of the approach presented here. Because there are two distinct variables in the model, there are two types of coupling forces: spin-coupling and activation-coupling, both of which can have a positive or negative valence. The generic effects of these coupling interactions are illustrated schematically in Figure 4 below. When two systems are attractively spin-coupled, their spin variables experience a force that acts to minimize their relative phase difference; when they are repulsively spin-coupled, the force acts to maximize their relative phase difference. When two systems are excitatorily activation-coupled, the systems act mutually to increase one another's activation; when they are inhibitorily coupled, they act to mutually decrease one another's activation. By hypothesis, attractive spin-coupling entails excitatory activation-coupling, and vice versa. The same entailments are not hypothesized to obtain between repulsive spin-coupling and inhibitory activation coupling, but excitatory coupling between two systems cannot co-occur with repulsive coupling, and inhibitory coupling cannot co-occur with attractive coupling. A further innovation is the use of a gating variable associated with each planning system, although the effects of this variable were implicitly present in [5]. The gating variables of inhibitorily coupled planning systems interact to prevent those systems from being co-selected.

The dynamics of activation in the model are responsible for selection of movement plans: inhibitory activation-coupling between plans, in combination with competitive gating variables, determines which plans are competitively selected. A important insight is that movement plans associated with onset consonants and vowels can be co-selected: their activation variables are not inhibitorily coupled and their gating variables are not competitive. In contrast, coda consonants are sequentially selected, because they exhibit inhibitory activation-coupling with other gestures and are competitively gated. A distinction is maintained from previous approaches [22], [34] between *planning systems*, which correspond to pre-motor plans for units such as gestures, syllables, and words, and *gestural (driving) systems*, which correspond to lower-level articulatory goals and can be represented as intervals in a gestural score in which motor commands drive articulator movement [19]. Furthermore, phase-coupled spin variables accomplish coordinative timing by mediating between the selection of planning systems and the activation of driving systems. The spin variables are intrinsically oscillatory, and the model maps the relative phases of co-selected systems into post-selection delays of movement initiation. In other words, after co-selection, spin determines precisely when driving systems are activated, and hence governs the precision control of movement initiation.

## Methods

### Selection with activation variables

Each gestural plan is associated with an activation variable *x*, indexed by *i*. The index *i* corresponds to a gestural or prosodic planning system, as determined by the lexical content of an utterance. For current purposes, the prosodic planning systems included in model simulations are syllables and words. There is a hierarchical relation between planning systems, such that each word system is associated with some number of syllable systems, and likewise each syllable system is associated with some number of gestural planning systems. These associations are determined by the lexical content of the utterance. Although both prosodic and gestural planning systems are selected, only gestural planning systems drive movement.

The dynamics of each activation variable are governed by a potential function *V_x_* and corresponding vector field -*dV/dx*, such that the time-derivative of *x* is equal to the negative of the derivative of its potential function with respect to *x*, plus a Gaussian stochastic noise term *η_x_* (Eq. 1). The activation potential (Eq. 2) is a function of three unit-specific variables: activation-coupling (*v*), gating (*w*), suppression (*y*), as well as activation (*x*) and intrinsic decay. The activation potential function can be conceptualized as a composite function that is the combination of several forces acting upon the activation variable. First, there is an intrinsic activation decay (*c_d_*). Second, there is a gating potential (*w*). Third, there is an activation coupling potential (*v*) which reflects inhibitory and excitatory interactions between units. Fourth, there is a suppression potential whose contribution to the vector field takes the form of a suppression-scaled exponential function of *x*, which is translated so that suppression is zero when *x* = 0.



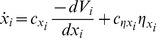
(1)




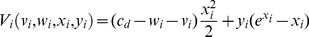
(2)


The gating variables *w*_i_ are limited to the range [0,1] and exhibit a constant growth or decay depending upon the selection of competing units (Eq. 3). When units *i* and *j* compete the sign of their activation coupling parameters is negative, i.e. X*_ij_* <0. The signs of the matrix X are symmetric but the magnitude of the coupling strengths need not be. The variable 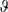
formalizes the notion of having a competitor selected: 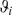 is the number of currently selected units which compete with *i*. Selection occurs when *x* is greater than an arbitrary threshold *τ*, here assumed to have a constant value, *τ*  = 1. Hence gating variables *w* will decrease to a value of -1 (fully closed) when competing systems are selected, and will increase to a value of 1 (fully open) when no other competing systems are selected. The growth/decay rates of the gating variables (*c_gate_*) are large so that gates open and close quickly.



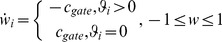
(3)




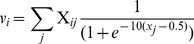
(4)

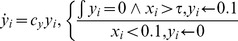
(5)


The activation coupling term is the summation of the influence of other units on *i* (Eq. 4). If the sign of the coupling strength *X_ij_* is negative, unit *j* exerts an inhibitory effect on *i*; conversely, if the sign of *X_ij_* is positive, *j* exerts an excitatory effect on *i*. A sigmoid function is used to describe the magnitude of the activation coupling force exerted by *j* on *i*; the input to the sigmoid is the activation of unit *j*, which is translated and rescaled so that an activation of 0 has a negligible influence and an activation of 1 has a maximal influence. The suppression variable is governed by Eq. (5); prior to plan selection suppression is initially 0 and hence exhibits no growth. When the activation of a unit first surpasses the threshold, the value of its suppression variable is set to 0.1, and begins to rise with a growth rate of c*_y_*. When the activation of that system falls below 0.1, the suppression variable is reset to 0.

**Figure 5:**
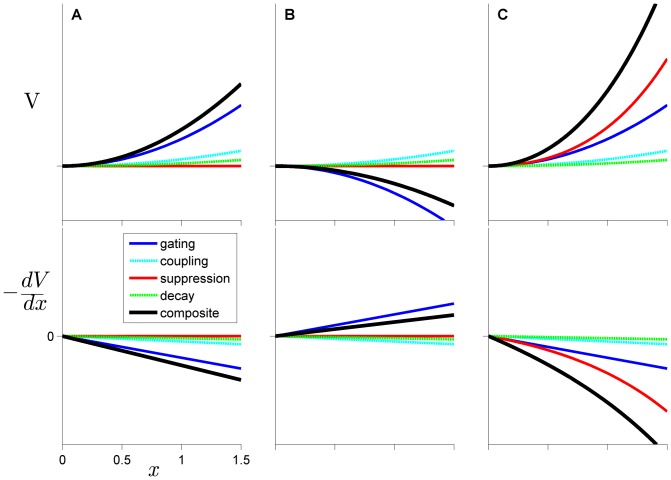
Activation potential functions and corresponding vector fields from three stages of articulatory production: (A) prior to response intention, (B) after response intention before selection, (C) after selection. Composite (black lines) and component functions (colored lines) are shown.

Figure 5 shows example potential functions in the top row, with functions representing the magnitudes and signs of the corresponding vector fields in the bottom row. The composite (black lines) and component (colored) functions are shown in each example. Figure 5A depicts a state in which the gating variable is closed, which would characterize a system prior to the intention to initiate movement. Since the slope of the gating potential is positive, the corresponding vector field is negative, and hence the potential exerts a force that diminishes activation. The same is true for the intrinsic decay of activation and in this case activation coupling forces. Figure 5B shows the potential when the gating variable opens as the result of an intention to produce a response. For the gating potential, x = 0 becomes an unstable equilibrium. The magnitude of the force exerted by the gating variable contributes more to the composite potential than inhibitory coupling forces or intrinsic decay, and hence activation will rise. Figure 5C shows the potential after recurrent inhibition triggered by selection has induced supression and the gating variable has closed, so that activation decays rapidly.

### Coordination with spin variables

The spin variables exert additional control over the precise timing of movement initiation by imposing delays on the activation of driving variables. The delays depend upon the phases of gestural systems relative to their associated syllable. The spin variables are modeled with a phase angle *θ_i_* which is 2π-periodic. The phase velocity (Eq. 6) is the sum of three components: an intrinsic frequency (*ω*), phase-coupling forces (derived from the relative phase potential *V_φ_*), and a Gaussian noise term *η_ω_*. Phase coupling forces are the negative derivative of the relative phase potential, with relative phase defined as *φ_ij_*  =  *θ_i_ – θ_j_*, following [55], [56]. The parameter *α_ij_* describes the phase-coupling force exerted by the spin variable of unit *j* on the spin variable of unit *i*. The signs of the matrix *α* are symmetric, but their magnitudes need not be. If *α_ij_* is positive, an attractive spin-interaction exists between *i* and *j*, and the stable equilibria of *Vφ_ij_* are located at *mod_2π_* (*φ_ij_*)  = 0. Conversely, if *α_ij_* is negative, a repulsive spin-interaction exists between *i* and *j*, and *mod_2π_* (*φ_ij_*)  =  π are the stable equilibria.



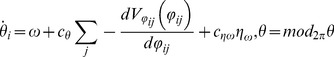
(6)




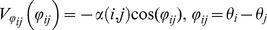
(7)


### Generating gestural scores

Gestural activation functions (which are distinct from planning activation) and a corresponding gestural score (see [19]) are obtained from driving variables (*D*). Only gestural planning systems are associated with driving variables, and furthermore, the control of driving variables is entirely feed-forward: they do not influence the activation, suppression, or gating variables of planning systems. The dynamics of the driving variables are grossly approximated by assuming that *D* is limited to the range [0,1] and receives a step increase when a delay variable *d* surpasses 0. When a unit has not been selected, the value of its delay variable *d* is 0 and the driving variable is quiescent. Upon selection (i.e. when activation becomes suprathreshold), *d* is set to the linearized relative phase upon selection (*φ_sel_*) and grows at a constant rate. The linearized relative phase upon selection *φ_sel_* is the difference between the phase of a unit and its corresponding anchoring unit. The anchoring unit for a gesture is assumed to be its associated syllable. The linearization is such that it maps phase differences in the interval [-π, π] to the interval [-π, 0], preserving the signs of unwrapped phase differences. When *d* exceeds 0, the driving variable *D* is activated with a unit step and subsequently decays at a rate of *c_D_*. Hence selection of a unit induces activation of the driving system after a brief delay, with the timing of movement initiation delayed from selection in proportion to *φ_sel_*. In this way, the coordinative interactions associated with spin exert an influence on precisely when movements are initiated.



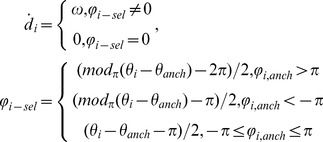
(8)




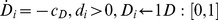
(9)


### Simulations and parameters

**Figure 6:**
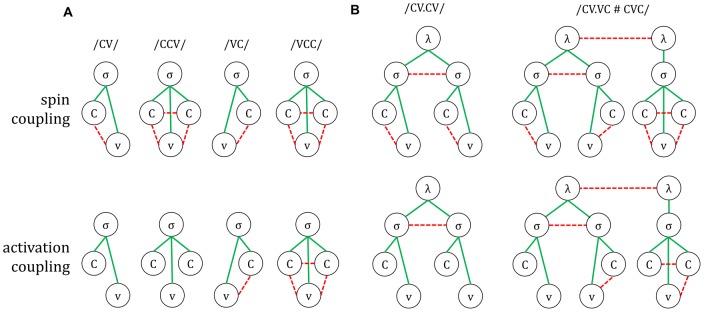
Examples of activation and spin coupling graphs. Attractive/excitatory coupling relations solid lines) and repulsive/inhibitory coupling relations (dashed lines) are shown. (A) monosyllables; (B) multisyllabic and multi-word responses.

Differences between response forms (e.g. between a/CV/ and/CCVCC/ syllable, or between a one and two-word response) correspond to systematic differences in the patterns of coupling interactions between component systems. A compact representation of these patterns is known as a *coupling graph* [22], [34], [36]. Coupling graphs shown in Figure 6 represent activation- and spin-interactions between planning systems, where attractive and excitatory coupling relations are indicated by solid lines, and repulsive and inhibitory relations are indicated by dashed lines. Figure 6A shows activation and spin coupling graphs for a variety of monosyllabic forms.

The coupling graphs are best understood in the context of a two-part generalization: when a pair of systems on the same level of the speech planning hierarchy interact, their activation and spin interactions will be inhibitory and/or repulsive, and when a pair of systems on different levels of the hierarchy interact, their interactions will be excitatory and attractive. An early version of this generalization has been called the *principle of like interaction* in [36]. For current purposes, we can view the speech planning hierarchy to consist of three levels: words, syllables, and gestures. Each unit in the speech plan is associated with its own planning system, which has both a spin variable and an activation variable. Not all the variables of simultaneously active system will interact, yet those interactions that do occur are important for planning and execution. Crucially, the nature of the interaction between a given pair of systems is constrained by the levels of the hierarchy with which those systems are associated.

According to the principle of like interaction, all between-level interactions are both excitatory and attractive. It follows that for a given syllable, all of its associated gestures will become more highly activated when the syllable becomes more highly active, and their spin variables will experience attractive forces acting to minimize their phase angles relative to the syllable's phase angle. It likewise follows that all within-level interactions will be inhibitory and/or repulsive. For articulatory gestures it is hypothesized that interacting consonantal and vocalic systems are repulsively spin-coupled, while only coda consonant gestures are inhibitorily activation-coupled (to each other and to vocalic gestures). Because inhibitory activation-coupling entails competitive gating, it follows that coda and vowel gestures cannot be co-selected, whereas onset and vowel gestures can be co-selected and their spin-coupling interactions can influence the timing of their initiation.

The principle of like interaction also obtains for prosodic systems such as syllables and words. Figure 6B shows coupling graphs for a disyllable/CV.CV/ and a two-word sequence. Syllable systems associated with the same word are inhibitorily coupled, and all word systems are inhibitorily coupled. In contrast, systems from different levels are excitatorily and attractively coupled if they are lexically associated. It is furthermore assumed that there are relatively low magnitude competitive coupling forces between heterosyllabic gestures and syllables associated with different words (these are not shown in the figure for purposes of clarity).

Since our primary interests are in the timing of response-initial movement initiation (i.e. reaction time) and the relative timing of consonant and vowel movements within syllables (i.e. onset/coda asymmetry), the values for parameters influencing movement durations are, for current purposes, of secondary concern. Moreover, the duration of time in which a gesture drives movement for a given speech sound is subject to many sources of systematic variation in natural speech. These include characteristics of the speech sound itself, speech rate, stress and boundary-adjacency [57], various forms of pragmatic and paralinguistic emphasis, as well as language-, dialect-, and speaker-specific variation. Hence there exists no “normal” movement duration for a given gesture. For practical purposes, the movement trajectories shown in Figure 1 were used as a guide. In those tokens the onset and coda consonantal movements last about 100–150 ms. The durations of vocalic gestural movements are longer and more variable (250–400 ms), since they depend to a larger extent on the preceding and following articulation(s). Note that the model controls the period of time in which gestural driving variables are active (greater than zero), whereas movement durations observable in kinematic data are commonly truncated due to gestural blending, which is determined in the task-dynamic model of [19] by a function that weights the contributions of simultaneously active gestures in the control of articulator movements. Here however, the decay rates of driving variables were selected by making the simplifying assumption that the durations of articulatory movements equate to the duration of time in which driving variables are active. To obtain these approximated movement durations, *c_D_*  = 10 for consonants and *c_D_*  = 5 for vowels (see Table S1 for further details).

Model equations were implemented as libraries in the Matlab Simulink environment, and simulations were run using the ode4 numerical solver and a fixed time step of 1 ms. Instantaneous frequencies ω of spin variables were set to 4 Hz, reflecting a typical syllable duration of 250 ms [58], [59]. The values of all other simulation parameters are reported in Table S1.

## Results and Discussion

This section presents the results of model simulations and compares them to empirical patterns. The empirical data considered here are speech reaction times and the relative timing of movement initiation in word onsets. It is shown that the model is able to simulate onset/coda timing asymmetries, relative timing of onset consonant and vowel gestures, effects of response preparation on reaction time, and effects of utterance composition (number of words and syllables per word) on reaction time. It should be noted that articulatory timing and reaction times exhibit variation from speaker to speaker and utterance to utterance; moreover, numerous experimental design factors or design-induced biases may contribute to this variation. The space of such factors is exceedingly large and mostly unexplored. Because of this, speech production models aim primarily to capture qualitative patterns in data. As long as a model is capable of simulating qualitative patterns, close fits to quantitative values of data can usually be easily obtained by selecting model parameters which optimize those fits. Hence even though the model can be parameterized to fit the data closely, assessment of the model should be based on its ability to simulate a variety of empirical patterns in a qualitative sense.

Second, all of the existing empirical data related to the effects of utterance composition on reaction time are derived from acoustic measurements. The large sample sizes required to detect such effects are generally prohibitive for articulatory studies, which are more time-consuming and expensive than acoustic studies. Acoustic reaction times are measured using an algorithm which detects the onset of acoustic energy associated with vocal fold vibration during vowels. This response detection scheme does not detect word-initial voiceless consonants that precede a vowel. Furthermore, it is well-known that articulatory movements in a syllable precede the onset of vocal fold vibration by a substantial amount that depends on the composition of the syllable onset [48], [49], [52]. Because the model simulates the initiation of articulator movements, there is necessarily a discrepancy between the model predictions and the empirical data derived from acoustic reaction times. This is not problematic because we are primarily concerned with qualitative similarity between the model and empirical data.

Although the activation-spin model is relatively more complex than a simple coupled oscillators model or competitive selection model, one benefit of this complexity is that the model can simulate a wider range of empirical patterns involving articulatory timing and movement initiation; an additional benefit is that the model offers a coherent explanation for a diverse group of phonetic and phonological asymmetries between onset and coda consonants, which neither coordination or selection mechanisms alone can account for. In spite of the complexity of the model, there are strong constraints on the hypothesized coupling parameters that govern model behavior: when between-level interactions are present, they are excitatory and attractive, and when within-level interactions are present, they are inhibitory and/or repulsive. This suggests there exists a more fundamental connection between the activation and spin variables and their coupling interactions, though the nature of that fundamental connection currently remains elusive.

### Simulation of onset/coda asymmetry

**Figure 7:**
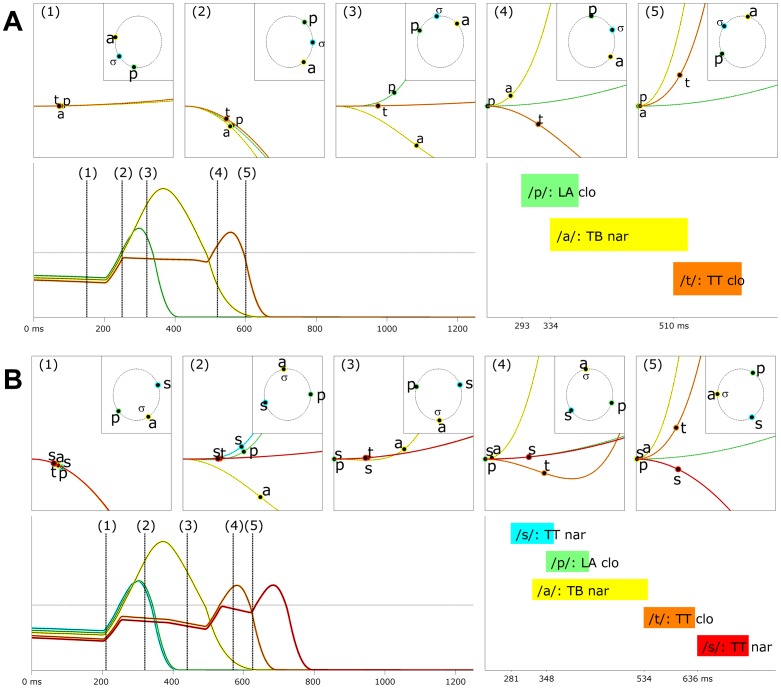
Simulations of onset/coda asymmetry and complex onsets/codas. (A) a CVC form “pot”. (B) a CCVCC form “spots”. Top rows of panels show potential functions and phases from a sequence of five stages in the planning of the utterance. Bottom rows of panels show activation variables and gestural driving functions.

The activation-spin model accounts for differences in how onset and coda consonantal movements are timed relative to vocalic movements. In the model, onset consonantal gestures are not inhibitorily coupled to each other or to vocalic gestures, and hence onset consonantal gestures and vocalic gestures can be co-selected. Furthermore, the timing of the initiation of onset and consonantal gestures is precisely coordinated by spin dynamics. In contrast, coda consonantal gestures are competitively selected relative to a preceding vowel or consonantal gesture. Figure 7A illustrates the selection and spin dynamics of articulatory plans in a CVC response, /pat/ “pot”. The top row of panels shows activation potential functions and planning system phases from five stages in the planning and production of the response. The stages were chosen for illustrative purposes and their times are indicated in the bottom left panel, which shows planning system activation. Prior to the initiation of movement (stage 1), the articulatory plans for each consonant are active but below-threshold; gating variables are closed, and hence activation exhibits a gradual decay (cf. the positive slope of the activation potentials). When the intention to produce the response is manifested, all gating variables are opened and activation levels rapidly increase (stage 2; cf. the negative slope of the activation potentials). When the/p/ and/a/ plans rise above the selection threshold, the gating variable of/t/ is closed (stage 3). The relative phase of the/p/ and/a/ spin variables at this time determines precisely when their articulatory plans will be initiated by imposing a relative-phase dependent delay on the activation of driving variables. In this same stage, suppression of/p/ and/a/ plans begins to grow and their activation levels decrease. When/p/ and/a/ activation fall below threshold, the gating variables are reopened (stage 4), and/t/ activation rises until this plan is also selected and subsequently suppressed (stage 5). The periods of time in which the driving variables are active is shown in the bottom right panel of Figure 7A.

The co-selection of the/p/ and/a/ allows for their associated gestures to be initiated with a delay of around 40 ms, whereas the competitive selection of the/t/ and/a/ leads to a more substantial delay between movement initiation (about 180 ms). Note that the order in which competitively interacting units are selected is determined by relative activation levels when gates are opened: the/p/ and/a/ plans were more highly active than the/t/ prior to the initiation of the response. This activation difference is assumed to be lexically specified, i.e. part of the long-term memory associated with the form. Figure 7B illustrates a more complex CCVCC form, in this case/spats/ “spots”. Here it can be observed that the onset consonant and vowel plans are co-selected, while both coda consonant plans are sequentially selected after the preceding plan is deselected. The difference between co-selection and competitive selection of a pair of plans arises from the setting of just a single parameter, activation-coupling, which in turn determines whether gating variables are competitive. Inhibitorily coupled plans are competitively selected, un-coupled plans can be co-selected.

Because the model allows for co-selected movement plans to be coordinated, it can simulate patterns of articulatory timing in complex onsets, in particular the c-center effect. Figure 7B shows that the initiations of the onset consonants in “spots” are displaced in opposite directions from the vocalic gesture: this timing pattern is attributable to the manner in which selection and coordination interact: once a gesture is co-selected, the precise moment of its initiation is delayed. The delay is determined by the phase of the gesture's spin variable relative to the spin variable of its associated syllable. Hence the coordinative interactions that influence spin variables determine the precise timing of movement initiation. In a complex onset, syllable-gesture spin interactions are attractive, acting to minimize the relative phase of consonantal and vocalic spin variables; these attractive forces oppose the action of the repulsive spin-coupling between gestures, which act to maximize their relative phase. Hence the equilibrium compromise between these opposing forces depends on the ratio of the magnitude of attractive syllable-gesture coupling forces to repulsive gesture-gesture coupling forces.

### Dependence of RT on preparation

The activation-spin model accounts for effects of preparation on reaction time through differences in initial activation between prepared and unprepared responses. As discussed above, reaction time to initiate an utterance depends on whether response plans have been prepared. The difference between RT in prepared and unprepared response tasks in the model is reflected by a difference in activation when selection gates are first opened. The reaction time to initiate a response is defined as the time from when gating variables are opened (representing an intention to initiate the response) to when the first articulatory driving variable rises above zero. For prepared responses, activation levels are relatively high when the gates are opened, and hence the initial plan reaches the selection threshold rapidly. For unprepared responses, activation levels are relatively low when gates are opened, and hence it takes longer for activation to grow to exceed the selection threshold. This additional delay in reaction time (ΔRT) can be conceptualized as a “retrieval period”, in which motor plans are being retrieved from long-term memory. Along with the activation level prior to gate-opening, the activation potential gain *c_x_* also influences how quickly activation grows and hence the time between the gate-opening and selection.

**Figure 8:**
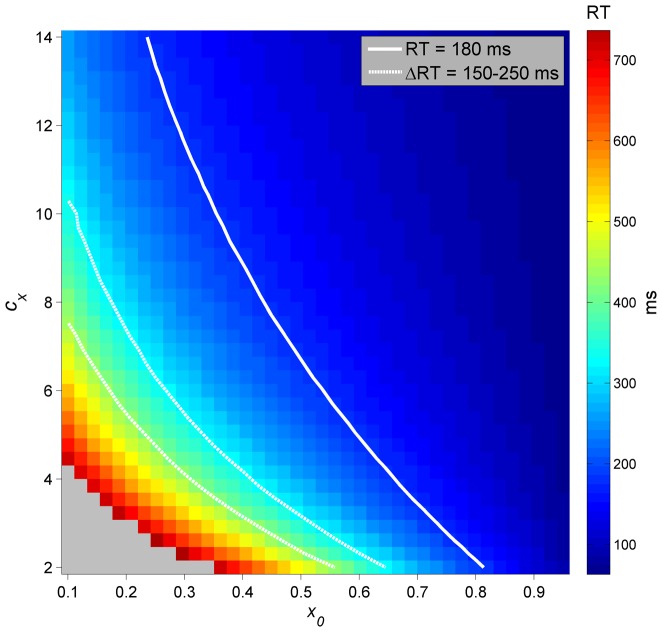
Dependence of RT on the activation potential gain (*c_x_*) and initial activation (*x*_0_). Bold line shows prepared-response RT  = 180 ms, dashed lines show corresponding range of ΔRT  = [150, 250] for a given value of *c_x_*.

Initial activation and the activation potential gain *c_x_* were varied in simulations of CV syllables in order to determine parameter values whose RT outcomes are consistent with behavioral findings for simple CV syllables in both prepared (delayed) and unprepared (immediate) response paradigms. Gating variables were opened one time step (0.001 s) into the simulation, so that the effects of activation decay are negligible and thus the initial activation is equivalent to the activation level at gate-opening. There are two parameters of interest: *c_x_* (activation gain) and *x*_0-prep_ (initial activation), and one outcome variable, RT. Hence the parameters resulting in a given RT form a trajectory in the parameter space. Furthermore, we are interested in *x*_0-unprep_ (initial activation in unprepared responses), which should result in ΔRT in the range of 150–250 ms. Figure 8 shows the dependence of RT on the activation potential gain (*c_x_*) and initial activation (*x*_0_). As *x*_0_ decreases, a constant RT of 180 ms (the empirical value in [48]) can be achieved by increasing *c_x_*. For a given *c_x_*, there is a range of unprepared-response *x*_0_ that result in ΔRT of 150–250 ms. Notice that this range expands slightly as prepared-*x*_0_ decreases. However, when prepared-*x*_0_ falls below about 0.45, it is not possible to achieve a ΔRT of 150–250 ms before initial activation of unprepared plans reaches zero.

Hence the model can successfully simulate the difference between prepared- and unprepared-response reaction times by manipulation of initial activation. However, the above results only obtain specifically for single-word, monosyllabic CV responses independent of variation in word structure. The RT values will also depend on the excitatory activation coupling between gestures and syllables, on coupling between syllables and words, on initial activations of those systems, and on inhibitory activation coupling forces that increase with utterance length and word complexity. In the following section we consider two of these sources of variation: effects of utterance length (number of words) and effects of word complexity (number of syllables in each word).

### Hierarchical effects on RT

The activation-spin model can be readily extended to incorporate hierarchical effects of syllables and words in utterance planning and execution. As discussed previously, it has been shown that the RT in prepared responses depends on both the number of syllables in the initial word of an utterance and on the number of words in the utterance. Moreover, these effects were observed to be linear and additive [39]. Simulations were conducted in which the number of words in a prepared utterance was varied from one to three and likewise the number of syllables in each word was varied from one to three. Hence a two-word, disyllabic utterance would have the form/CV.CV # CV.CV/, while a three-word, trisyllabic utterance would have the form/CV.CV.CV # CV.CV.CV # CV.CV.CV/. All parameters for a given type of system were identical across simulations and independent of position in the utterance (see Table S1), with the exception of initial activations. Initial activations of syllables were set to a constant percentage of the initial activation of their associated word; likewise, initial activations of gestures were a constant percentage of the initial activation of their associated syllable – this ensures that in the absence of large noise fluctuations, words, syllables, and gestures are selected in the intended order.

**Figure 9:**
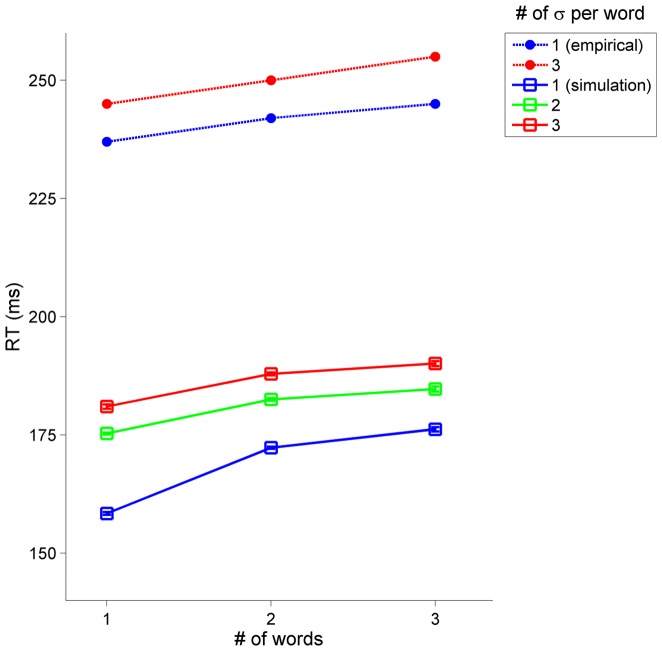
Dependence of RT on the number of words (utterance length) and the number of syllables per word (word complexity). The magnitudes of length and complexity effects are comparable in model simulations (squares) and empirical results (circles).

Model-simulated effects of utterance length and word complexity on RT are quite comparable to the empirical pattern. Figure 9 shows simulated RT as a function of the number of words in the utterance and the number of syllables in each word (squares), along with empirical data from [39] for comparison (filled circles). The large discrepancy between simulated and empirical baseline values follows from the fact that the empirical data are based on acoustically measured RTs, whereas the simulated RTs are based on the initiation of articulatory movement. The key correspondence between model and empirical data pertains to the sizes of the length and complexity effects. Excepting the relatively short RT of a one-word monosyllable, the effects between other forms are quite in line with the empirical results. For example, the empirical effect of word length has a slope of about 10 ms/word, and the simulated results are nearly the same for disyllabic and trisyllabic words. The empirical effect of word complexity is such that RT increased by 12 ms between monosyllabic and trisyllabic forms. In simulations this value is about 16 ms and 18 ms for two- and three-word utterances, respectively. Hence model simulated RTs accord fairly well with empirical observations. This behavior of the model is due to the inhibitory activation coupling between planning systems on the same level of the hierarchy. Syllables inhibit each other, resulting in a word complexity effect; the same holds for words, which results in the utterance length effect. In both cases, the presence of a greater number of inhibitory interactions diminishes activation of prosodic systems and thereby reduces the extent to which they augment gestural activation; this in turn results in a longer period of time for gestural activation to exceed the selection threshold.

### Accounting for onset/coda asymmetries

The success of a model can be judged by its ability to provide a unified understanding of a group of apparently unrelated phenomena. Phonetic and phonological differences between onsets and codas are good candidates for a group of phenomena which a theory of speech motor control should explain. A number of such differences were listed in section 1, and are presented again in Table 1 below. The coupled oscillators model hypothesizes that coda consonantal gestures are anti-phase coordinated with a preceding gesture, and hence attributes some of these patterns to the relative instability of anti-phase coordination. In contrast, the activation-spin model associates coda consonantal gestures with selection events that are distinct from selection of preceding gestures. Here we evaluate how the two approaches – anti-phase coordination and competitive selection – compare in accounting for the patterns, and conclude that the concept of competitive selection in the activation-spin model offers a more comprehensive understanding of differences between onsets and codas.

**Table 1:** Comparison of the standard coupled oscillators model and the activation-spin model in accounting for onset/coda asymmetries.

	Coupled oscillators model	Activation-spin model
restricted combinatoriality	✓	✓
templatic constraints	-	✓
tonal capacity influence	-	✓
stress location influence	-	✓
compensatory lengthening	-	✓
instability in repetition	✓	?

In many languages, the distribution of coda consonants is more restricted than that of onset consonants: onset consonants combine relatively freely with vowels, while coda consonants may be prohibited from occurring with certain vowels or may be altogether disallowed in coda position. The coupled oscillators model attributes the greater propensity for restrictions on codas and dependency between codas and vowels to the relative instability of anti-phase coupling of coda consonants [7], allowing for individual combinations to be made more stable through learning. In contrast, the activation-spin model attributes these patterns to a greater likelihood of overlap between a coda consonantal gesture and onset gestures from a subsequent syllable, which is ultimately attributable to the independence of coda gestural selection. When performing multiple selection events in association with a syllable, gestures associated with the last selection process are more likely to obscured by gestures associated with a following syllable. This can occur because the following syllable is selected relative to the preceding syllable, not preceding gestures.

Selection events proposed by the activation-spin model can be related in a fundamental way to moraic theory and constraints on the minimal forms of morphemes/syllables [9], [12]. Many phonological analyses posit the existence of a level of subsyllabic structure known as the “mora,” where the number of moras associated with a syllable often has consequences for a diverse range of patterns involving word/syllable shape, tone, and stress. A short vowel in the syllable nucleus constitutes one mora, long vowels and diphthongs constitute two moras, and in many languages coda consonants are held to be associated with a mora. Prohibitions on codas are more common when the preceding syllable nucleus is bimoraic, and this is typically viewed as a constraint against trimoraic syllables. It is not clear in the context of the coupled oscillators model why codas are substantially more restricted after bimoraic syllable nuclei. But, if we hypothesize that a bimoraic vowel involves two selection events, then the prohibition on codas after bimoraic vowels can be seen as a constraint against producing three selection events in association with a syllable. In a sense, this hypothesis entails that a diphthong is bimoraic because the component vowel gestures each occupy a selection event, and a long vowel is bimoraic because the vocalic gesture is selected twice. The trimoraic syllable constraint follows naturally from the model, since gestures associated with the last selection event in a syllable are more likely to be obscured by gestures associated with the following syllable. Indeed, exceptions to constraints against three tautosyllabic selection events are most commonly observed in word-final syllables, where coda consonantal articulations are least prone to being obscured by those of a following syllable.

For another example of how selection can inform our understanding of moraic theory, consider that some languages require a morpheme to have at least two moras, such that bimoraic CVV, CVC, and CV.CV morphemes are allowed while a monomoraic CV is not [9]. This sort of requirement can be interpreted as a requirement that a word involve at least two selection events. It is not clear how the distinction between in-phase and anti-phase coupling can account for this sort of minimality constraint – it provides no obvious way to draw an inherent connection between a CVC morpheme and CVCV morpheme. In contrast, the activation-spin model provides a straightforward way of understanding such restrictions and relating them to restrictions on coda combinatoriality.

Another set of phenomena that can be readily understood in relation to selection events involves prosodic features such as tone and pitch accents, which have recently been conceptualized as gestures and have been shown to interact with consonantal and vocalic articulatory gestures [60], [61]. In languages with lexical tone, syllables with codas or long vowels (i.e. bimoraic syllables) often have a greater capacity to bear complex or contour tones, i.e. tones which consist of multiple pitch targets. The activation-spin model straightforwardly accounts for this observation because it holds that a bimoraic syllable has an additional selection event in which an additional tonal gesture can be co-selected and coordinated. Along the same lines, in languages with stress, the presence of a bimoraic syllable often influences the location of stress, such that stress will occur on the first bimoraic syllable relative to the beginning or end of a word. Because intonational pitch accents always associate with stressed syllables, the attraction of stress to bimoraic syllables can be viewed to result from the availability of an additional selection event in which a component of an intonational pitch accent gesture can be selected. In contrast, the hypothesis that codas are anti-phase coordinated with a preceding vowel does not seem to offer a straightforward explanation for why stress can be influenced by syllable shape.

Compensatory lengthening is likewise another phenomenon that falls out naturally from the activation-spin model, but which is not easily amenable to explanation on the basis of coordination alone. When a coda consonant is lost in the course of diachronic sound change, it is sometimes observed that the preceding vowel lengthens, apparently preserving the bimoraicity of the syllable; this same compensatory lengthening does not occur when an onset consonant is lost [14]. This asymmetry in phonological behavior has been taken as evidence that onset consonants always share a mora with the following vowel. The distinction can be related as above to the hypothesis that coda consonantal gestures are associated with an independent selection event, whereas onset consonantal gestures are co-selected with the following vowel. If it is hypothesized that the selection event associated with a coda can be replaced by re-selection of the vocalic gesture, then the preservation of moraic structure observed in compensatory lengthening can be understood via selection mechanisms. In contrast, the anti-phase coordination mechanism proposed by the coupled oscillators model would be forced to interpret compensatory lengthening as a situation in which a vocalic gesture becomes anti-phase coordinated with itself, a situation which is somewhat undesirable theoretically.

The last asymmetry listed in Table 1 less conclusively differentiates the two models considered here. In accelerating repetition tasks, a VC syllable often reorganizes into a CV syllable at a sufficiently high repetition rate [15], [16]. The coupled oscillators model offers a conceptually appealing account of this reorganization: the variation of a control parameter (rate) causes the relatively less stable anti-phase coordination employed for VC to become unstable, inducing a phase-transition to the in-phase coordination of CV. This can moreover be related to similar observations in nonspeech domains [21]. To account for this, the activation-spin model must stipulate that the phase-transition is accompanied by a loss of inhibitory interaction between the consonantal and vocalic gesture, allowing for co-selection. Exactly why rate acceleration would induce this loss of inhibitory interaction is not immediately evident, although one possible mechanism might involve habituation.

In sum, the activation-spin model offers a more comprehensive unified explanation for a variety of phonetic and phonological patterns involving onset/coda asymmetry. In particular, it provides an intuitive explanation for morpheme/syllable minimality and maximality constraints, interactions between tone/stress and syllable structure, and compensatory lengthening. A purely coordination-based model does not readily account for these patterns. Moreover, the activation-spin model provides an equally plausible account of the relative prevalence of restrictions on codas, while maintaining a coordination-based account of articulatory timing in syllable onsets.

## Conclusion

The activation-spin model was shown to successfully account for important behavioral phenomena involving the timing of speech articulation. These include: (1) onset-/coda-vowel timing asymmetry, (2) coordinative timing patterns in simplex and complex onsets, (3) the dependence of reaction time on utterance preparation, and (4) effects of utterance length and word complexity on reaction time. More importantly, the model offers a unified explanation for a diverse collection of phonetic and phonological asymmetries. Here these findings and their implications are discussed further. In addition, we consider potential neurological grounding and future elaborations of the model.

The model was successful in replicating the asymmetry in consonant-vowel timing between onset and coda consonants. Onset consonantal gestures and vocalic gestures are known to be initiated close together in time. The model simulates subtle temporal effects involving the coordination of syllable-initial gestures by means of a compromise between repulsive spin interactions among consonantal gestures and attractive spin interactions between consonantal gestures and syllables. Coordination of word-initial consonants and vowels is observed in a number of languages where there is a c-center effect that such the consonantal gestures in a C_1_C_2_V form appear to be temporally displaced in opposite directions from the vocalic gesture. For example, evidence of this effect has been observed in English [23], [24], French [62], Georgian [63], and for most clusters in Italian [26]. Here we have shown that this pattern can be understood to arise from the absence of inhibitory coupling/competitive gating among word-initial consonants and the following vowel. In contrast, there are some languages in which the immediately prevocalic consonant of a complex onset is not displaced when additional consonants occur initially, i.e. the timing of the prevocalic consonant relative to the vowel does not differ between CCCV, CCV, and CV forms. This has been found to be the case in Moroccan Arabic [64], Slovak [65], Tashlhiyt Berber [66], [67], and for/sC/ clusters in Italian [26], where non-prevocalic word-initial consonants are not syllabified as onsets. This pattern can be understood to arise when all consonants except for the prevocalic one are sequentially selected and hence do not influence the coordinative interactions between the prevocalic consonant and vowel.

The notion that the selection and coupling interactions of onsets and codas differ is important because of its explanatory potential for a number of phonological patterns related to syllable structure. Like the coupled oscillators model, the activation-spin model can account for the restricted combinatoriality of coda consonants. In addition, its use of competitive selection for coda consonants allows for a number of phonological patterns to be understood. These include minimality/maximality constraints on morpheme/syllable shapes, influences of syllable structure on tone and stress, and compensatory lengthening patterns.

In addition to accounting for gestural timing patterns, the model captures reaction time phenomena related to utterance preparation, utterance length, and word complexity. In all of these cases, the role of activation is key. Reaction times in prepared utterances are faster than those in unprepared responses because initial activation levels are higher in the former, and hence it takes less time for the activation of the initial movement plan to reach the selection threshold. The effects of utterance length and word complexity arise from inhibitory interactions between activation variables: when there are more plans, each plan experiences more inhibition and so it takes longer for the initial plan to reach the selection threshold. This holds for word systems and for syllable systems within words. A similar explanation is feasible for related effects not specifically modeled here. For example, reaction times are shorter in production tasks for more frequent words and syllables [46], [47], [68]. This could arise if the frequency of a word or syllable is associated with a higher-level of initial activation.

It should be noted that the activation-spin model is highly nonlinear and incorporates many parameters; thus there is a risk that the model may be overparameterized, allowing for spuriously accurate fits of the data. However, the quantitative values of empirical speech data depend on factors such as task design and measurement procedure, the possibilities of which have been only sparsely sampled experimentally. For this reason, a more appropriate assessment of the model is with regard to its qualitative correspondence with empirical patterns. In this regard, overparameterization is less of a concern, because a model cannot produce a good qualitative correspondence with a range of data if it does not have an adequate structure. This of course begs the question of whether the model structure is motivated in any way.

On that note, many aspects of the model can potentially be related to neural mechanisms or other aspects of motor control. The intent in describing these relations here is not to make definitive claims about neural mechanisms, but rather, to shed light on elements of the model which might otherwise seem entirely arbitrary, and to suggest ways in which the activation-spin model might be further extended or grounded in neurophysiology. For one, the intrinsic frequency of planning systems (in simulations above: ω  = 4 Hz, T_ω_  = 0.250 s) was chosen for consistency with typical syllable durations in speech, but notably this value also corresponds to the low end of the range associated with cortical theta rhythms, 4-8 Hz. Theta-band power has been observed in MEG and EEG recordings in premotor cortex and has been argued to provide a coordinative mechanism for motor execution [69]–[72], to facilitate the maintenance of plans in working memory [73], and to regulate sensorimotor integration [74], [75]. Although theta oscillations have been ascribed diverse functions and their exact role in motor planning remains unclear, it is possible that spin dynamics are instantiated through neural mechanisms that give rise to these low-frequency oscillations.

Another aspect of the activation-spin model that can be related to neural systems involves the relation between activation, gating, and selection. The gating variables serve to prevent the simultaneous selection of competing active plans. In this regard, the relation between activation and gating is analogous to the relation hypothesized to exist between the frontal cortex and the basal ganglia: motor plans are actively maintained in pre-frontal/pre-motor cortex and are selectively disinhibited by the basal ganglia, which results in selection of motor responses [76]–[78]. The structure of the model implies that articulatory plans associated with different segmental units need not be competitively gated. This is certainly true within a given segment, where several gestures are often coordinated. For example, the segment [n] in the word “nap” involves the co-selection of three gestures: a tongue-tip constriction gesture to create a closure against the palate, a velum lowering gesture to allow air to flow through the nasal cavity, and a glottal adduction gesture to enable vibration of the vocal folds. The model asserts that the same co-selection can occur between gestures associated with different segments.

The role of suppression variables in the activation-spin model is to decrease activation after a plan has been selected. This relation between activation and suppression may be associated with models of feedback control in which motor activation is modulated by a mismatch between sensory target representations and a motor efference copy [79]–[83]. For example, in the hierarchical state feedback control model of [79], activation of abstract lexical and conceptual representations actives corresponding pre-motor representations as well as sensory representations of expected somatosensory and auditory feedback corresponding to the motor actions. When active, the sensory representations exert excitatory influences upon the motor representations, but the motor representations inhibit corresponding sensory ones. These motor-to-sensory inhibitory connections are hypothesized to serve the function of a forward model, i.e. a representation of the expected consequences of motor actions expressed in sensory coordinates. As the motor plans become highly active, they inhibit the sensory representations and hence diminish their own activation. In other words, when the state feedback model observes a match between target sensory feedback and anticipated sensory feedback, it turns off motor activity. The dynamics of the suppression variables in the activation-spin model may be seen as a manifestation of this process. Furthermore, the model can potentially be extended to operate with state feedback control by incorporating sensory target representations and sensory-motor interactions for each articulatory gesture.

One open issue in the model regards the nature of articulatory targets and how those targets are mapped to movements of individual articulators. Here we assumed the task-dynamic model [19], in which the activation of a gesture displaces an equilibrium expressed in coordinates of vocal tract geometry; the motion of a gestural system to the new equilibrium drives the movement of individual articulators through a weighted inverse model that relates tract geometry to articulator positions. However, there are viable alternative control frameworks, such as threshold control theory [84]–[86], or forward-inverse neuromotor computation [87], [88] that can accomplish the control of movement trajectories. Since the activation-spin model addresses the relatively high-level processes of motor selection and initiation, it could potentially be used to govern any of these control schemes.

Future development of the activation-spin model should also attempt to model the effects of diverse sources of variation on kinematic properties such as movement durations, velocities, and targets. One challenge in addressing this is that empirically observed movement durations do not correspond precisely to the durations of time in which the model's gestural driving variables are active. Specifically, due to gestural blending and the overlap of gestures sharing the same articulatory effectors, the durations of physical movements will be truncated relative to the period of time in which their associated gestures are active [19]. In the model presented here, movement durations follow simply from the decay rates of driving variables, but there are many factors which interact with movement plans to influence durations. Among these are the articulatory context in which a movement occurs, speech rate, rhythm, and proximity to prosodic boundaries. One possibility that warrants consideration is that suprathreshold gestural planning activation may modulate gestural driving variables, such that driving variables decay more slowly when gestural planning activation is higher, as might be the case for gestural planning systems associated with rhythmic or prosodic prominence. Such ideas have been considered in [53], [54], but have not yet been integrated into the present model.

In sum, this paper investigated a dynamical model of sequential speech movement planning. It was argued that sequencing and coordination of movement arise from distinct cognitive mechanisms associated with two dynamical variables, activation and spin. In combination with a competitive gating mechanism and a selection threshold, activation regulates the ordered selection of movements. Spin variables oscillate and their relative phases exert precision control over the timing of the execution of co-selected movements, such as tautosyllabic onset consonants. Because selection and coordination are general phenomena, this model can be applied to non-speech domains of motor control. The model presents the first unified dynamical approach to investigating timing phenomena across several levels of the speech planning hierarchy, from words to syllables to articulatory gestures. Moreover, the model draws a novel connection between moraic theory and selection mechanisms, associating moras with selection events. This enables the model to provide a unified explanation for a diverse body of linguistic phenomena.

## Supporting Information

Table S1 **Model parameters used for simulations of hierarchical coupling shown in** **Figure 9**.
(DOCX)
