# Supplementary material for: A Dynamical Model of Hierarchical Selection and Coordination in Speech Planning
Source: PLoS One. 2013 Apr 24;8(4):e62800. doi: 10.1371/journal.pone.0062800 (PMC3634742; doi:10.1371/journal.pone.0062800)
Supplement: Table S1 — Model parameters used for simulations of hierarchical coupling shown in Figure 9 . (DOCX) [file pone.0062800.s001.docx]

| **Table S1.** Model parameters used for simulations of hierarchical coupling shown in Figure 9. | |
| --- | --- |
| Gating variables are initially set to -1, and are opened at t = 0.010 s by setting them to 1. Abbreviations: (g) gestural planning system; (C) consonantal gestural planning system; (V) vocalic gestural planning system; (σ) syllable planning system; (W) word planning system | |
|  |  |
| Unit parameters |  |
| *ω* = 4 Hz | Frequencies of all spin variables are 4 Hz (period = 0.250 s) |
| *c_x_* = 8 (g), = 20 (σ,W) | Activation growth rate |
| *c_y_* = 20 (g, σ), = 4.5 (W) | Suppression growth rate |
| *c_θ_* = 10 | Spin potential gain |
| *c_ηx_* = 0.01 | Activation noise gain |
| *c_ηω_* = 0.01 | Spin noise gain (*c_ηω_* = 0 for the RT simulations in Figure 9) |
| *c_d_* = 0.1 | Activation decay rate |
| *c­_D_* = 5 (V, σ, W), = 10 (C) | Driving variable decay rate |
| *c_gate_* = 100 | Gating variable growth rate (>>1 so that gates open and close rapidly) |
|  |  |
| Coupling parameters (*α_ij_*, *X_ij_* : effect of system *i* on system *j*) | |
| *X*_gg_ = 0 (co-selected)  *X*_gg_ = -0.01 (competitive) | Gestural activation coupling: co-selected gestures are not coupled, e.g. onset consonants and vowels; competitive gestures are inhibitorily coupled, e.g. vowels and coda consonants. |
| *X*_σσ_ = -0.15 | Syllable activation coupling: all syllables are inhibitorily coupled. |
| *X*_ww_ = -0.05 | Word activation coupling: all words are inhibitorily coupled. |
| *X*_σg_ = 0.10 (within-σ)  *X*_σg_ = 0 (between-σ)  *X*_gσ_ = 0 | Gesture-syllable activation coupling: syllables exert an excitatory force on associated gestures. |
| *X*_wσ_ = 0.10 (within-W)  *X*_wσ_ = 0 (between-W)  *X*_σw_ = 0 | Syllable-word activation coupling: words exert an excitatory force on associated syllables. |
| α_gg_ = -1 (within-σ)  α_gg_ = 0 (between-σ) | Gestural spin coupling: all gestures within a syllable are repulsively coupled. Negligible spin coupling between heterosyllabic gestures. |
| α_σσ_ = -1 (within-W)  α_σσ_ = 0 (between-W) | Syllable spin coupling: all syllables within a word are repulsively coupled. Negligible spin coupling between hetero-word syllables. |
| α_ww_ = -1 | Word spin coupling: all words are repulsively coupled. |
| α_σg/gσ_ = 1.6 | Gesture-syllable spin coupling: syllables and gestures are attractively coupled, symmetrically. |
| α_wσ_ = 1.6  α_σw_ = 0 | Syllable-word spin coupling: words exert an attractive force on syllables, but not vice versa. |
|  |  |
| Initial conditions (for simulations of hierarchical coupling in Figure 10) | |
| *x_0_*(*W_i_*) = [0.8, 0.6, 0.4] | Initial activation of word systems |
| *x_0_*(σ*_ij_*) = [0.9, 0.85, 0.80]  × *x_0_*(*W_i_*) | Initial activation of syllable systems, parameterized as a percentage of initial word activation. |
| *x_0_*(g*_ijk_*) = [0.9, 0.85]  × *x_0_*(*σ_ij_*) | Initial activation of gestural systems, expressed as a percentage of initial syllable activation. |
| *θ_0_* = 0 (σ, W)  *θ_0_* = [0.6 -0.6] (CV) | Initial phases (in radians). |
